# Development of molecular markers for genetic male sterility in *Gossypium hirsutum*

**DOI:** 10.1007/s11032-015-0336-z

**Published:** 2015-06-07

**Authors:** Xuehui Feng, Don Keim, Humphrey Wanjugi, Issa Coulibaly, Yan Fu, John Schwarz, Scott Huesgen, Seungho Cho

**Affiliations:** Monsanto Company, 800 N. Lindbergh Blvd, St. Louis, MO 63141 USA

**Keywords:** Genetic male sterility (GMS), Hybrid cotton, Molecular markers, Haplotype, Phenotype prediction

## Abstract

Genetic male sterility (GMS) in cotton mediated by two homozygous recessive genes, *ms5ms5* and *ms6ms6*, is expressed as non-dehiscent anthers and unviable pollen grains. Sequence analysis on *ms5* and *ms6* loci in *Gossypium hirsutum* was conducted to reveal genomic variation at these two loci between GMS and wild-type *G. hirsutum* inbred lines, and sequence polymorphism linked to *ms5* on A12 and *ms6* on D12 was revealed. A haplotype marker set that consisted of four SNPs targeting both *ms5* and *ms6* gene regions was developed and validated for association with GMS in cotton. Predictability of GMS phenotype by this haplotype SNP set was over 99 %. GMS haplotype marker set can serve as a high-throughput molecular breeding tool to select GMS individuals and improve hybrid production efficiency.

## Introduction

Genetic male sterility (GMS) in cotton occurs in a form of non-dehiscent anthers and unviable pollens when reproductive development process fails due to possible loss of nuclear gene functions required for pollen development. Unlike cytoplasmic male sterility, genes related to GMS phenotypes can be easily transferred to various genetic backgrounds by routine crossing practice and allow the trait to be inherited at full penetrance in successive generations. There have been 19 different GMS genes, *Ms1* to *Ms19*, identified in tetraploid cotton species (summarized by Chen et al. 2009), and are classified by genetic characterization and multigenic nature such as single dominant gene (*Ms4*), single recessive gene (*ms2*) and paired recessive genes (*ms5* and *ms6*). Single recessive genes *ms1* and *ms3* reportedly conferred partial genetic male sterility (Justus and Leinweber 1960; Justus et al. 1963). In case of *ms3*, it produced fertile flowers more often in greenhouse compared with field condition (Justus et al. 1963). In contrast, GMS conferred by a single recessive gene, *ms2*, and paired recessive genes, *ms5* and *ms6*, conferred stable male sterility (Richmond and Kohel 1961). Because GMS maintainers need to be fertile, recessive genetic mechanisms with complete male sterility especially mediated by *ms5* and *ms6* have been widely used in hybrid cotton production (Basu 1996).

Cytoplasm male sterility (CMS) technology is also available to produce hybrid cotton. The first cytoplasmic male sterility was developed by transferring diploid A2 genome of *Gossypium arboreum* to cytoplasm of *Gossypium anomalum* carrying diploid B1 genome. Male sterile plants were produced when CMS inducer was pollinated by pollens of *G. arboreum*, and fertility was restored with *G. anomalum* pollen (Meyer and Meyer 1965; Meyer 1969). Because of incomplete or unstable male sterility and undesirable traits in CMS lines generated by *G. arboreum* and *G. anomalum* cytoplasm system, alternative CMS system called CMSD-2 was created by transferring genomes of commercial cotton (*G. hirsutum*) to *Gossypium harknessii* cytoplasm (Meyer 1973, 1975). Restorer of CMSD-2 system was created by transferring a restorer gene, *Rf1*, in *G. harknessii* to *G. hirsutum* genome (Meyer 1975). Cytoplasm of *Gossypium trilobum* carrying diploid D8 genome was also used to develop CMS system known as CMS-D8 (Stewart 1992), and *Rf2* in D8 genome of *G. trilobum* was found to restore fertility (Zhang and Stewart 2001). *Rf1* functioning sporophytically was found to be nonallelic to *Rf2* which functions gametophytically, and these two genes were linked on chromosome LGD08 which is same to D05 (Zhang and Stewart 2001, 2004; Liu et al. 2003; Feng et al. 2005; Wang et al. 2007; Yin et al. 2006). In spite of high male sterility frequency close to 100 % by CMS, broader application of CMS to hybrid production has been limited due to narrow genetic choices of CMS and restorer line combination and cytoplasmic effect on potential yield drag and CMS stability (Meyer 1969; Meyer and Meyer 1965; Bhale and Bhat 1990; Schnable and Wise 1998).

The sources of *ms5* and *ms6* genes were speculated to be *G. tomentosum* or progeny from an interspecific cross with *G. hirsutum*. A nectariless F_3_ individual from a cross of Stoneville 20 × nectariless *G. tomentosum* was crossed with a paternal parent, Empire WR. From this cross, a nectariless F_3_ individual was crossed with Gregg. An F_2_ individual with nectariless phenotype was crossed to Lankart 57 and a single male sterile individual with nectariless phenotype was identified in their F_2_ progenies (Weaver 1968). Recessive genetic inheritance of GMS trait was confirmed by backcrossing fertile progenies from the sterile individual against paternal ancestors. Assuming that there has been no spontaneous mutation occurring during crossing and population advancement, emergence of male sterility within F_3_ progenies from interspecific cross between *G. hirsutum* and *G. tomentosum* might be due to interaction between two genes originated from two different tetraploid species. The recessive allele of either *ms5* or *ms6* originated from *G. tomentosum* might have interacted with the recessive allele that already existed in *G. hirsutum* to express male sterility. Although unlikely, the chance of spontaneous mutation at any stage throughout the interspecific and intraspecific crosses cannot be ruled out and can be considered as a possible source of GMS until disproven.

Genetic map positions of GMS genes were revealed by linkage mapping using SSR markers (Chen et al. 2009). Two Chinese GMS source lines, Lang-A carrying a recessive gene, *ms15*, and Zhongkang-A carrying two recessive genes, *ms5* and *ms6*, were crossed to Hai7124 carrying homozygous dominant alleles of both *ms5* and *ms6* first. Their F_1_ progenies were backcrossed to sterile parents to generate mapping populations in which only one of the two genes was segregating. Authors mapped each gene independently to chromosomes 12 (*ms5* and *ms15*) and 26 (*ms6*) and claimed that *ms5* and *ms15* are two different genes on chromosome 12 with a distance of 6 cM between loci. Rhyne (1991) reportedly mapped *ms8* and *ms9* on chromosome 12 and 26, respectively, as well. It is uncertain whether they are related to *ms5* and *ms6* as a test cross between these two genetic sources has not been attempted.

Although molecular and biochemical mechanisms of male sterility caused by *ms5ms5* and *ms6ms6* are not fully understood, analogous studies conducted in other species could be used to predict potential mechanism(s) that cause pollen development failure in cotton. In *Arabidopsis*, causes of failure in male gamete development were classified into four cases: (1) non-functioning stamen resulting in deformed anthers, (2) failure in microsporogenesis, (3) lack of pollen-coating agent, tryphine, and (4) non-dehiscent anthers (Okada and Shimura 1994). Observation of stamen and pollens from male sterile flowers indicated that all four cases appear to contribute to the sterility phenotype in cotton. Additional molecular mechanisms related to pollen development and mutant phenotypes due to genetic mutations on metabolic pathways are well characterized in model species (reviewed by Wilson and Zhang 2009; Okada and Shimura 1994; Chaudhury 1993). Genes of other functions but which are still associated with pollen development in *Arabidopsis* have been well characterized at the molecular level which include the following: *MS1*, a PHD-finger family of transcription factor related to microsporogenesis (Wilson et al. 2001; Ito et al. 2007), *MYB* transcription factor related to dehiscence of pollen and pollen wall development (Steiner-Lange et al. 2003; Preston et al. 2004), *MS2*, elongation/condensation complex presumably related to pollen wall formation (Aarts et al. 1997), *AtPTEN*, tumor suppressor homolog related to pollen cell death after mitosis (Gupta et al. 2002). With in-depth whole-genome sequence data available, reverse genetic approaches can be used to further identify candidate genes associated with genetic male sterility in cotton. To characterize the genetic and genomic characteristics of the GMS in cotton, we conducted genomic analysis at *ms5* and *ms6* loci. Our study revealed genomic variation at both loci and also within candidate genes possibly associated with genetic male sterility in cotton. By SNP markers designed to detect genomic variation at both loci, a haplotype for GMS and predictability of GMS phenotype by SNP haplotyping were determined.

## Materials and methods

### Genetic characterization of GMS

Segregating populations for genetic study of GMS were generated from a cross between DPGh98018 and DPGh04651. DPGh98018 was used as a full fertile male parent, and DPGh04651 was used as a female parent presumably carrying multiple recessive GMS genes. Multiple recessive genetic inheritance patterns of GMS in DPGh04651 were observed within progenies generated by multiple generations of selfing in nursery in Scott, Mississippi during 2004–2005 season (data not provided). Male sterility of each plant was determined by monitoring presence of non-dehiscent anther in five flowers at anthesis over a month. In addition, flower abortion was also observed to confirm male sterility. Plants were scored as fertile if at least one dehiscent anther was visible. The same method was applied to phenotype all genetic materials used in our study. Based on inheritance patterns of GMS within progenies of DPGh04651, two recessive genes presumably *ms5* and *ms6* were speculated to confer GMS in DPGh04651. All genetic materials used in our study were prepared based on GMS mechanism by paired *ms5* and *ms6* genes. Sibcrossing between fertile and sterile F_2_ progenies from a cross between DPGh98018 and DPGh04651 was made to produce sibcross F_1_ progenies in the greenhouse in 2004. Sibcross F_1_ progenies were planted in greenhouse, and male sterility was phenotyped by each sibcrossing event. Fertile F_1_ progenies were advanced to sibcross F_2_ populations by selfing. Segregation pattern of male sterility was monitored in each sibcross F_2_ population independently, and sibcross F_2_ populations segregating for male sterility were selected for further genetic study.

### SNP discovery by amplicon sequencing of *ms5* on A12 and *ms6* on D12

Two recessive GMS gene, *ms5* and *ms6*, were previously mapped to chromosomes 12 (A12) and 26 (D12), respectively, using SSR markers (Chen et al. 2009). The primer sequences of SSR markers linked to *ms5* and *ms6* were mapped to diploid D genome sequence of *G. raimondii* (JGI v2.0, annot v2.1, www.cottongen.org) by in silico comparative mapping approach. The diploid D genome sequence between 19,114,325 bp and 21,205,044 bp was used to discover genomic polymorphism linked to *ms5* and *ms6*. PCR assays to produce sequencing templates were designed using Primer3 (http://frodo.wi.mit.edu/primer3/) with default parameters. *G. hirsutum* lines including TM-1, DPGh98018 (male fertile) and DPGh04651 (GMS donor) and diploid A (*G. arboreum*, PI629477) and D (*Gossypium raimondii*, PI530898) as subgenomic references were used for amplicon PCR. To demonstrate homoeology between two loci on chromosome 12 and 26, respectively, chromosome nomenclature of A12 and D12 was used instead.

### PCR for sequencing and SNP genotyping

PCR to amplify sequencing template was prepared using Platinum^®^ Taq DNA Polymerase High Fidelity manufactured by Life Technologies (Grand Island, NY) following the manufacturer’s manual. The PCR was conducted by initial denaturation at 95 °C for 5 min followed by 35 cycles of denaturation at 95 °C for 1 min, annealing at 55 °C for 30 s, and extension at 72 °C for 2 min and a final extension at 72 °C for 5 min. Sequencing of PCR amplicons was performed using 3730XL DNA Analyzer (Life Technologies, Grand Island, NY), and sequence analysis and SNP discovery were conducted using CLC Bio Genomics Workbench software (Aarhus, Denmark). All genomic polymorphisms identified by amplicon sequence analysis were converted to TaqMan^®^ assays for further SNP validation and SNP genotyping. DNA extraction was done by a protocol developed by Dellaporta et al. (1983).

PCR of 5 µl total volume for SNP assay validation and SNP genotyping was conducted using GTXpress™ Master Mix for SNP genotyping assay manufactured by Life Technologies following the manufacturer’s manual (Life Technologies, Grand Island, NY). Each PCR included two PCR primers (final concentration of 900 nmol for each primer), two TaqMan^®^ MGB probes with NFQ labeled by FAM or VIC dye (final concentration of 250 nmol for each probe) and 4 ng genomic DNA. The PCR was conducted using GeneAmp^®^ PCR System 9700 (Life Technologies, Grand Island, NY) by initial denaturation at 95 °C for 10 min followed by 40 cycles of denaturation at 95 °C for 15 s, annealing and extension at 60 °C for 60 s. SNP data collection and analysis were conducted by ViiA™ 7 Software v1.2.1. (Life Technologies, Grand Island, NY).

### Genetic linkage of SNPs to *ms5* and *ms6*

F_3_ populations were generated by combining 14 different sibcross F_2_ populations originated from a cross between DPGh98018 and DPGh04651. Genetic linkage between SNPs and chromosome assignment of linked markers to A12 or D12 were estimated by genetic distance calculated using R-Genetics (http://cran.r-project.org/web/packages/genetics/genetics.pdf) among newly developed markers and also to the chromosome-specific markers assigned to A12 and D12 (Cho et al. 2014).

Fine mapping of both *ms5* and *ms6* was carried out by association analysis using a population of 1044 inbred lines representing 899 Monsanto germplasm lines of diverse genetic backgrounds consisting of 880 male fertile and 164 male sterile individuals. Diverse genetic materials were generated by unstructured random mating using male sterile progenies originated from DPGh04651 and diverse Monsanto germplasm lines. Male sterile phenotypes for each plant were binary coded as 1 for male fertile and 0 for male sterile and used in association analysis. Each plant was also genotyped by SNP markers putatively linked to *ms5* and *ms6*, and two-step analysis was performed to identify haplotype marker set predicting male sterility. First, logistic regression was performed using binary sterility outcome with corresponding marker genotypes as predictors on the assumption of monogenic recessive model. As a second step, markers showing linkage to *ms5* or *ms6* loci were subjected to logistic regression analysis assessing relationships of alleles between two loci in association with the male sterility.

Haplotype SNPs associated with GMS were also tested on Lankart 57 (PI528822), Gregg (PI529094), Empire WR (PI529224) and *G. tomentosum* (PI 530723) which were reportedly served as donors of recessive *ms5* and *ms6* alleles (Weaver 1968), and the origins of recessive *ms5* and *ms6* alleles were estimated based on haplotype SNP patterns in each test material.

## Results

### Genetic characterization of *ms5* and *ms6* genes using mapping populations

Three independent sibcrossings between three different pairs of fertile and sterile F_2_ siblings produced sibcross F_1_ progenies, and fertile-to-sterile phenotype ratios within these sibcross F_1_ populations were close to 1:1 (Table 1). Based on 1:1 phenotype ratio within these small populations of sibling crosses, genotypes of fertile parent plants used in these crosses were speculated to be *Ms5ms5ms6ms6* or *ms5ms5Ms6ms6*, while sterile progenies were homozygous recessive for both loci. Fertile progenies from these sibcrosses were advanced to F_2_ populations by selfing, and male sterility phenotypes were monitored. Segregation patterns of phenotypes in F_2_ populations were either 1:3 sterile to fertile indicating that a single gene was segregating or 1:15 sterile to fertile indicating that both genes were segregating in each F_2_ population (Table 1).

**Table 1:** Segregation of genetic male sterility in F_1_ of F_3_ sibling crosses and their F_2_ progenies

Generation	Fertile frequency	Sterile frequency	Expected ratio (F:S)	Chi-square probability
F_1_^a^	11	19	1:1	0.144
F_1_^a^	1	2	1:1	0.564
F_1_^a^	3	0	1:1	0.083
F_2_^b^	51	27	3:1	0.05
F_2_^b^	26	16	3:1	0.05
F_2_^b^	25	11	3:1	0.441
F_2_^b^	76	18	3:1	0.19
F_2_^b^	45	20	3:1	0.283
F_2_^b^	399	35	15:1	0.118

^a^Sibcross F_1_ progenies from a cross between fertile and sterile siblings selected from F_3_ segregating populations

^b^F_2_ progenies from F_1_ by selfing

### Sequencing to discover SNPs for *ms5* and *ms6*

Whole-genome sequence of *G. raimondii* D genome (JGI v2.0, annot v2.1, www.cottongen.org) was used as a reference to nominate target regions for discovery of SNPs. Presumably about 0.5-Mbp-long sequences were subjected to amplicon sequencing. Sequence polymorphism was identified in forms of single-nucleotide polymorphism or single- or multiple-nucleotide deletion among the test materials which included male fertile and male sterile inbred lines on diploid A and D genome species. Among all genomic polymorphism identified within target regions covered by amplicon sequences, 23 polymorphic sequences qualified for PCR assay design were subjected to further analysis.

### Genetic linkage of SNPs to *ms5* and *ms6*

**Fig. 1:**
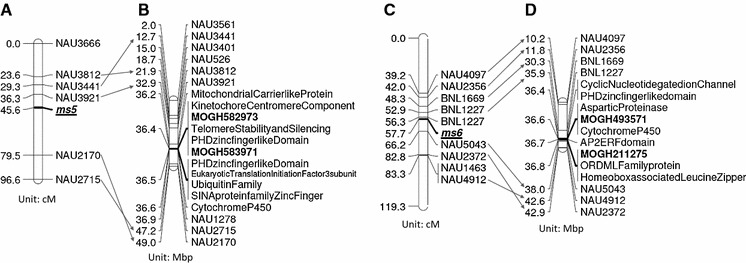
In silico comparative mapping of molecular markers linked to *ms5* and *ms6* to chromosome 08 of *G. raimondii* D genome (D5). Maps **a** and **c** were recreated from the publication by Chen et al. (2009) describing map positions of *ms5* and *ms6* on chromosome 12 (A12) and 26 (D12), respectively. Maps **b** and **d** are summary of in silico comparative map positions of SSRs and newly developed SNPs linked to *ms5* and *ms6* on chromosome 08 of D5 genome. In silico map positions of SSRs on D5 genome are available in CottonGen (http://www.cottongen.org/gb/gbrowse/JGI_221_Dgenome/). Gene names on maps **b** and **d** represent annotation of the template sequences used to develop SNPs

SNPs were tested for linkage to *ms5* on A12 and *ms6* on D12 using F_3_ populations originated from 14 different sibcross F_2_ populations segregating for male sterility. Polymorphisms of SNP markers corresponding to all 23 sequence polymorphisms identified by amplicon sequencing were tested in F_3_ populations, and genetic distances between SNPs and linkage to *ms5* and *ms6* were calculated based on genetic linkage to chromosome-specific SNPs. All 23 markers segregated in F_3_ sibcross populations, and all were assigned to *ms5* and *ms6* loci. Annotation of the putative genes carrying SNPs linked to *ms5* and *ms6* and their in silico comparative map positions on chromosome 08 on *G. raimondii* D genome (D5) are summarized in Table 2 and Fig. 1. Further analysis to associate each marker to the trait was conducted by the association mapping approach.

**Table 2:** Summary of amplicon sequences used to identify genomic polymorphism at *ms5* on A12 and *ms6* on D12 and their in silico comparative map positions on D5 genome of *Gossypium raimondii*

Chromosome	Sequence annotation	Position on chromosome 08 of D5 genome syntenic to A12/D12 (base pairs)^a^	Polymorphism
A12	Cytochrome p450	36601681—36601890	C/T
A12	Cytochrome p450	36601598—36601781	A/G
A12	Cytochrome p450	36600316—36600453	A/G
A12	SINA protein family (zinc finger)	36517055—36517264	A/G
A12	Ubiquitin family	36513917—36513713	A/G
A12	Eukaryotic translation initiation factor 3 subunit	36487438—36486837	C/T
A12	PHD-zinc-finger-like domain	36448804—36448648	A/G
A12	PHD-zinc-finger-like domain	36448539—36448899	C/T
A12	PHD-zinc-finger-like domain	36444422—36444671	C/T
A12	Telomere stability and silencing	36437252—36437058	C/T
A12	Telomere stability and silencing	36436860—36436639	C/T
A12	Kinetochore centromere component	36404821—36405030	C/G
A12	Mitochondrial carrier-like protein	36180644—36180843	G/T
D12	Homeobox-associated leucine zipper	36829091—36829251	C/T
D12	ORDML family protein	36823054—36823710	C/G
D12	AP2(ERF domain)	36670553—36670417	C/G
D12	Cytochrome P450	36600741—36600580	A/G
D12	Cytochrome P450	36600741—36600580	A/G
D12	Aspartic proteinase	36590566—36590776	A/G
D12	PHD-zinc-finger-like domain	36443655—36443785	G/C
D12	Cyclic nucleotide-gated ion channel	36432550—36432377	C/T
D12	Cyclic nucleotide-gated ion channel	36432269—36432083	C/G
D12	Cyclic nucleotide-gated ion channel	36429252—36429459	C/G

^a^Physical position of amplicon sequences on whole genome of *Gossypium raimondii* (JGI assembly v2.0, www.cottongen.org)

### Association mapping to *ms5* and *ms6* loci

Monsanto germplasm lines of diverse genetic backgrounds consisted of 880 male fertile and 164 male sterile inbred lines were genotyped by all 23 SNPs assigned to *ms5* and *ms6*. Male sterility phenotypes were collected from all association test materials and the phenotype data were categorized in binary format as either male sterile or male fertile. Using logistic regression model, all possible SNP combinations between two loci were tested to explain male sterility within diverse test materials. The recessive *ms5* allele was explained best by TT allele on MOGH583971 and TT allele on MOGH582973, and the recessive *ms6* allele was explained best by CC allele on MOGH211275 and GG allele on MOGH493571. When these two haplotypes for recessive alleles of *ms5* and *ms6* were combined, phenotype predictability in the diverse test panel was 99.6 % (Table 3). By putative gene annotation, these markers are located on PHD-zinc-finger-like domain protein on A12 and *AP2* gene on D12. Detailed SNP information for TaqMan genotyping is summarized in Table 4.

**Table 3:** Probability of male sterility occurrence using genotype combinations of MONGH583971 (TT) and MONGH582973 (TT) associated with *ms5ms5* and MONGH211275 (CC) and MONGH493571 (GG) associated with *ms6ms6*

Frequency (count)	Individual with male sterile haplotype on *ms5* and *ms6*	Individual with male fertile haplotype on *ms5* and *ms6*	*p* value	Probability
Male sterile individual	109	17	2.00E−16	99.60 %
Male fertile individual	1	769

**Table 4:** Haplotype SNP set designed to predict genetic male sterility in *Gossypium hirsutum* conferred by paired recessive genes, *ms5* on A12 and *ms6* on D12

Chromosome	SNP name	Polymorphism^a^	TaqMan SNP assay oligo sequence^b^
			(5′→3′)
A12	MOGH583971	C/T(*ms5*)	F: AGAACATTGATTATTACTGCCCCGAAT
			R: GGCTCTCTTTTTGCTAAGCATGATT
			P-FAM: CTTGGACTTCACTTTAC
			P-VIC: CTTGGACTTCGCTTTAC
A12	MOGH582973	C/T(*ms5*)	F: CCGGTTCGGTTTATAAGAGAAGATCTG
			R: AAATGTCTTTCTCTTCCTCGGAACC
			P-FAM: CTCTCACTCAATACTC
			P-VIC: ACTCTCACTCGATACTC
D12	MOGH211275	C(*ms6*)/G	F: AGCTAATGCTCTCTGACGGAACTA
			R: ACAAAAGAGTAGATTTTAGTGTACCGTGTATTT
			P-FAM: TGTGCGTAATTCATGATC
			P-VIC: TTGTGCGTAATTGATGATC
D12	MOGH493571	A/G(*ms6*)	F: CTCTCGGGCCTGAACGA
			R: GCTTTTACATCCCGGCTAAGACA
			P-FAM: TGGGACGACGTTGAA
			P-VIC: ATTATGGGACGATGTTGAA

^a^Single-nucleotide polymorphism linked to *ms5* and *ms6* was indicated by “*ms5* or *ms6*” designation

^b^F, R, P-FAM and P-VIC represent forward primer, reverse primer and two probes labeled by FAM and VIC dyes, respectively. Oligo sequences for SNP markers and template sequences of *ms5* and *ms6* are available at CottonGen (http://www.cottongen.org/search/markers)

### Origin of *ms5* and *ms6*

Lankart 57 (PI528822), Gregg (PI529094), Empire WR (PI529224) and *G. tomentosum* were tested for the presence of *ms5* and *ms6* alleles using the haplotype markers described above. Based on haplotype patterns of the samples tested, *G. tomentosum* indicated the presence of *ms5* and *ms6* and Lankart 57 indicated the presence of *ms6*. All other varieties tested showed no presence of the *ms5* and *ms6* haplotypes. From these results, it is probable that the *ms5* was transmitted from *G. tomentosum.* However, since none of the F_2_ populations occurring before the crossing with Lankart 57 showed male sterility (Weaver 1968), it is unlikely that the *ms6* allele from *G. tomentosum* was causative for male sterility. Therefore, the Lankart 57 *ms6* gene is most likely responsible for male sterility in one of its F_2_ progenies in combination with *ms5* from *G. tomentosum*. It is also possible for the *ms5* allele of Lankart 57 to have differential phenotypic expression when compared to the *G. tomentosum* allele.

## Discussion

Map positions of *ms5* and *ms6* were defined by Chen and his colleagues in 2009 using higher density SSR markers tested on backcrossing populations. By testing molecular marker patterns in *Ms5ms5Ms6ms6* of male fertile and *ms5ms5ms6ms6* of male sterile progenies, the authors were able to identify markers explaining dominant and recessive alleles of each gene. Markers found to be associated with the genes were further tested for genetic distance against other existing molecular markers. Using populations segregating for only one of the two genes with the second fixed to recessive homozygote is another effective approach beyond synthetic backcrossing population method used by Chen and his colleagues. However, the single gene segregating population approach will require tedious monitoring of progeny phenotypes in each family and can be very difficult to determine which gene is segregating in each population. In our study, we scanned entire genetic regions neighboring *ms5* and *ms6* and discovered markers polymorphic in diverse genetic backgrounds. All newly developed SNPs were quickly mapped to *ms5* and *ms6* by in silico comparative mapping using D diploid genome sequence and by linkage analysis using randomly segregating F_3_ populations. Based on known genetic locations and distances between markers, all possible pairwise combinations of markers between two different chromosomes were tested for association with male sterility using logistic regression modeling. As a result, two haplotypes consisting of 2 SNPs linked to *ms5* and 2 SNPs linked to *ms6* were identified that explained male fertility and sterility with 99 % accuracy.

Two GMS genes, *ms5* and *ms6*, were expected to be homoeologs and possibly duplicated through polyploidization. However, we found haplotype markers representing *ms5* and *ms6* located about 150 Kbp apart from one another on the diploid D genome sequence. If genomic variation exist only on one of two homoeologs, then it is still possible to identify polymorphic markers unique to one homoeologous chromosome. Additional genomic polymorphisms at flanking regions from the other homoeologous chromosome can be used as flanking markers and allowing the two haplotypes high phenotypic prediction accuracy.

From the in silico comparative mapping of template sequences of the SNPs to diploid D genome, genomic regions targeted by male sterility haplotypes appeared to be very narrow and contained a limited number of genes. Based on putative gene annotation, PHD-zinc-finger-like domain protein on A12 and *AP2* (ERF domain) on D12 were contained between markers defining the haplotypes. *ArabidopsisMALE STERILITY1* gene was found to be PHD-zinc-finger-like domain protein which plays a critical role in pollen development (Wilson et al. 2001; Ito et al. 2007). Recessive alleles created by EMS mutation conferred male sterility in *Arabidopsis* only when it is homozygous recessive. Pollens of *ms1* mutant with homozygous recessive alleles failed developing viable pollen (Wilson et al. 2001). The function of *APETALA2* (*AP2*) as a negative regulator of *AGAMOUS* has been well established using *Arabidopsis *flower development mutants. Stamen, ovule and other floral organ development was significantly prohibited in the *ap2* mutant (Jofuku et al. 1994). Further investigation is needed to prove functional association of any of these genes to male sterility in cotton.

The initial *ms5* and *ms6* study hypothesized the origin of the alleles from Gregg and Lankart 57. The reasoning is that following the identification of the first sterile individual, backcrossing was performed with Gregg and Lankart 57, each producing F_2_ segregating populations with 3:1 male-fertile-to-male-sterile ratio. Our study with haplotype markers tested on Lankart 57, Gregg, Empire WR and *G. tomentosum* showed that recessive *ms5* allele was present only in *G. tomentosum* and that recessive *ms6* allele was present in *G. tomentosum* and Lankart 57. This indicates that *ms5* allele in male sterile F_2_ progeny was likely transmitted from *G. tomentosum. H*owever, due to absence of male sterile progenies in the F_2_ populations occurring before the crossing with Lankart 57, it is likely that *ms6* allele causative for male sterility is found in Lankart 57 and not *G. tomentosum.* Although genotype test materials we used might not be identical to the ones used in the study by Weaver in 1968, the results from our study were able to explain potential sources of genetic male sterility in cotton.

In conclusion, a haplotype SNP marker set that consisted of four SNPs, two linked to *ms5* on A12 and two linked to *ms6* on D12 in *G. hirsutum* was developed. This haplotype marker set was able to predict male sterile phenotype at the rate of 99 % accuracy within our diverse germplasm test set. Each haplotype SNP marker can differentiate zygosity of *ms5* and *ms6* loci and can be used to predict GMS phenotype in *G. hirsutum* of diverse genetic background at any generations including inbred parents and progenies segregating randomly. With reliability as molecular markers and tight linkage to *ms5* and *ms6*, our GMS haplotype SNP marker set can serve as a high-throughput molecular breeding tool to select GMS individuals to be used as hybrid parents and also to confirm purity of hybrids produced through larger scale commercial seed production. This new molecular breeding tool should be able to eliminate tedious hand emasculation and fertile plant removal after flower phenotyping in the field and improve genetic purity and quality of hybrid production.
